# Minimally Invasive Evaluation and Treatment of Colorectal Liver Metastases

**DOI:** 10.1155/2011/686030

**Published:** 2011-07-07

**Authors:** Anton L. Gueorguiev, Richard Mackey, Gopal C. Kowdley, Jesus Esquivel, Steven C. Cunningham

**Affiliations:** ^1^Department of Surgery, Saint Agnes Hospital, 900 Caton Avenue, Mailbox no. 207, Baltimore, MD 21229, USA; ^2^Department of Surgery, Saint Joseph Hospital, Baltimore, MD 21231, USA

## Abstract

Minimally invasive techniques used in the evaluation and treatment of colorectal liver metastases (CRLMs) include ultrasonography (US), computed tomography, magnetic resonance imaging, percutaneous and operative ablation therapy, standard laparoscopic techniques, robotic techniques, and experimental techniques of natural orifice endoscopic surgery. Laparoscopic techniques range from simple staging laparoscopy with or without laparoscopic intraoperative US, through intermediate techniques including simple liver resections (LRs), to advanced techniques such as major hepatectomies. Hereins, we review minimally invasive evaluation and treatment of CRLM, focusing on a comparison of open LR (OLR) and minimally invasive LR (MILR). Although there are no randomized trials comparing OLR and MILR, nonrandomized data suggest that MILR compares favorably with OLR regarding morbidity, mortality, LOS, and cost, although significant selection bias exists. The future of MILR will likely include expanding criteria for resectability of CRLM and should include both a patient registry and a formalized process for surgeon training and credentialing.

## 1. Introduction

As the third commonest cancer in males and females and the second commonest cause of cancer death [1], colorectal cancer (CRC) is an important health problem in the world.

There are estimated to be 334 000 new cases of CRC in Europe [2] and 142 570 new cases in the United States [1], and 36% of these patients will succumb to their CRC [1]. Of all patients with CRC, approximately 65% develop distant metastasis, and the commonest location (40%) is the liver [3]. The proportion of patients who presented with synchronous versus metachronous colorectal liver metastases (CRLMs) in France was equal in a recent epidemiologic study: the proportion of patients with synchronous CRLM and the 5-year rate of metachronous CRLM were both 14% [4]. Unfortunately, only 25% of patients with CRLM are amenable to curative-intent treatment [3, 5–7]. 

Since the first report of a laparoscopic liver resection (LR) in 1992 (for CRLM) [8, 9], the field of minimally invasive liver resection (MILR) has seen tremendous advances, paralleling those of open liver resection (OLR), with increasing safety and efficacy. Although most early MILRs were for benign disease, the first report notwithstanding, an increasing volume of nonrandomized data suggests no oncologic disadvantage to performing MILR compared to OLR.

## 2. Minimally Invasive Evaluation

Transabdominal US is a widely available, inexpensive, and noninvasive technique of evaluating for CRLM but, compared with other modalities, has the lowest sensitivity and negative predictive value [10], excepting contrast-enhanced ultrasound, which by some studies is as sensitive as CT [11] but which is not available in the United States. Consequently, CT and MRI are among the most commonly employed modalities used to evaluate the liver for CRLM. A recent meta-analysis of diagnostic imaging of CRLM, evaluating 39 articles (3391 patients), found MRI to be the optimal first-line modality, with a per-patient sensitivity of 88% [12]. 

While early (1990s) studies showed staging laparoscopy (SL) to be superior to preoperative imaging in detecting unresectable or extrahepatic disease, thereby sparing as many as 34% of patients a laparotomy [13], when combined with intraoperative ultrasound (IOUS), even in an era of markedly improved axial imaging (2000s), SL was able to detect unresectable disease and potentially prevent unnecessary laparotomies in 10–25% of patients with primary and secondary hepatic malignancies [14–16].

Increasing quality in preoperative axial imaging technology, however, has challenged the use of laparoscopic IOUS. In a prospective study of 194 patients undergoing LR for CRLM published in 2008, Tamandl et al. compared data from preoperative imaging using multidetector CT (MDCT) and MRI to intraoperative findings using IOUS and bimanual palpation and found that IOUS provided useful information regarding additional CRLM in only 2.6% of patients [17]. Other groups, however, have consistently found that 10% of additional small tumors that were missed by axial imaging may be detected [18, 19] and the preoperative treatment plan may change in nearly half of cases [15] when a complete evaluation is performed, including exposure of the entire surface of the liver and porta hepatis, an IOUS scan of all 8 liver segments, porta hepatis and the paraceliac nodal bed, and a thorough evaluation of the entire peritoneal cavity to detect extrahepatic metastases. Whether done laparoscopically or open, a complete US evaluation of the liver should include four steps: (1) an identification of intrahepatic vascular anatomy, (2) identification and characterization of known lesions, (3) a search for previously unrecognized lesions, and (4) the planning of a treatment strategy, which may include resection, ablation, or both. Intrahepatic tumors are evaluated for size, number, location, relationship to biliary and vascular structures, and echogenicity (most CRLMs are hypoechoic (42%) or isoechoic (43%), while a minority (15%) are hyperechoic [18]). Echogenicity should be noted because it has been shown to correlate with long-term survival: in a prospective evaluation of 147 patients at Johns Hopkins Hospital, the 5-year survival for patients with hypo-, iso-, and hyperechoic CRLM was 14%, 37%, and 46%, respectively [20].

## 3. Minimally Invasive Treatment Techniques

### 3.1. Ablative Techniques

Standard ablative techniques include both chemical and thermal ablation and are increasingly used, both in isolation for patients with unresectable CRLM and in combination with LR. Chemical ablation with ethanol or acetic acid has been performed for CRLM but is less effective for CRLM than for hepatocellular carcinoma [21–23]. Thermal ablation is therefore the preferred treatment for CRLM not amenable to surgical resection. Thermal ablation includes radiofrequency ablation (RFA), microwave, laser, and cryoablation. In these techniques, focal heating or freezing of tumor cells causes local tumor destruction while preserving surrounding hepatic parenchyma. An emerging and still poorly studied technique is the nonthermal, nonchemical technique of electrical ablation using irreversible electroporation.

Although operative ablation of CRLM is often done in combination with both MILR and OLR, percutaneous ablation of CRLM is appropriate in cases in which no resection is planned and offers an attractive minimally invasive treatment option for such patients. However administered, RFA is relatively safe and less invasive than formal hepatic resection, but several notable complications may occur, including hepatic failure, hydrothorax, intraperitoneal bleeding, hepatic abscess, bile duct leaks, and tumor seeding [24–28]. The reported procedure-related morbidity ranges from 2% to 12% and the mortality rate from 0% to 4.3% [24, 26–29]. Many of these reports, however, include a variety of approaches, including percutaneous, laparoscopic, and open, and include more cases of HCC than CRLM [24, 27–29]. Given the frequent association of HCC with cirrhosis, complications such as bleeding, liver failure, and death may be more common following ablation of HCC than CRLM [24, 27]. In a recent report of 100 patients undergoing RFA for CRLM, there was no procedure-related morality and the major complication rate was 8% [30]. Another study of 100 patients undergoing RFA (146 treatments) for CRLM revealed a major complication rate of 4.8%, including 1 death from liver failure [31]. Gillams and Lees similarly found a major complication rate of 4.0% in a series of 167 patients undergoing RFA for CRLM [32].

Microwave ablation (MWA) is an emerging technology that can also be performed open, percutaneously, or laparoscopically. Like with RFA, however, no randomized trials support its use over other techniques. The primary theoretical advantages of MWA are the ability to ablate larger lesions and to do so faster. These advantages likely derive from the fact that MWA, unlike RFA, does not rely on electric current and so is not impeded by tissue desiccation and charring, both of which decrease electrical conductivity. In addition, there is no so-called “heat sink” effect, or heat loss from adjacent blood vessels, which in the case of RFA decreases the effectiveness and increases the time required for ablation. Most reported experiences have demonstrated safe and effective MWA, with complications and local recurrence rates comparable to RFA [33–35]. Theoretical disadvantages include the inadvertent injury to adjacent structures and the risk of such collateral damage makes tumor location important in the decision of which modality (e.g., RFA versus MWA) and route (e.g., open versus laparoscopic) to choose. Ablation of dome lesions or left lateral segment lesions, for instance, could expose the diaphragm and heart to thermal injury and serious morbidity, and such lesion may be better treated with an open or laparoscopic as opposed to percutaneous technique. 

Cryoablation can similarly be performed via percutaneous or open approaches. It offers many of the same benefits of RFA and MWA, such as preservation of liver parenchyma, but at a potentially increased cost, given that the complication rates may be higher compared with RFA: morbidity 10–40% and mortality 0–5% [36]. Complications include hepatic/iceball fracture (19%), hemorrhage (3.7%), coagulopathy (3.8%), biliary fistula (2.9%), and organ failure [36]. Furthermore, animal models have demonstrated that there is a more severe systemic response following cryotherapy than following RFA [37]. 

Irreversible electroporation (IRE) is an emerging, nonthermal, nonchemical technique that uses electrical current to destroy cells. Its reversible counterpart, reversible electroporation, is a common laboratory technique that has been employed for decades to transiently render cells widely permeable to allow entry of large molecules such as drugs and genes by applying an electrical field to cells that are otherwise not permeable to the molecules of interest [38]. IRE, previously viewed as an undesireable upper limit of reversible electroporation because it rendered the cells permanently permeable and therefore nonviable, is now used clinically as a form of nonthermal ablation. While supported by animal models [39–41] and available commercially for use in patients, IRE has not been well studied in patients. It does have the theoretical advantages, however, of being very fast (micro- to milliseconds), preserving connective tissue architecture thereby allowing ablation close to vital structures in the liver hilum for example, and unlike thermal techniques it is not likely affected by blood flow [38]. As with many new techniques, all cases of IRE performed should be registered to document the role and safety of IRE, as well as to identify important questions for study in clinical trials, and indeed such a registry is underway at the University of Louisville [42]. 

### 3.2. Ablation and Resection

1. Surgical resection is the standard of care for CRLM, but RFA has produced comparable outcomes for limited disease, with some important caveats. Although RFA has demonstrated 5-year survival rates as high as 30% in some studies [31, 32] and numerous other studies have attempted to compare resection and ablation [43–47], recent analyses using propensity score methodology [48, 49] have shown that comparison of survival rates following RFA and resection is not reliable, due to major differences in clinicopathologic characteristics, RFA technologies and expertise, and analysis of margin status (namely, lack of pathologic analysis after RFA since no specimen is available). Until randomized controlled trials comparing RFA and surgical resection are available, the question of their comparability will likely remain unanswered [50]. In a recent systematic review performed by the American Society of Clinical Oncology in 2009 regarding RFA for CRLM [51], over 400 published articles were reviewed, 46 identified as having over 10 patients with adequate followup and perioperative data to analyze. There was a wide variability in the reported 5-year survival (14–55%) and local tumor recurrence (3.6–60%) [51].

While comparisons to resection may be unreliable and largely unrevealing, comparisons within RFA series have been more fruitful. For instance, it has become clear that increasing CRLM size—especially size >3 cm—is directly proportional to shorter survival and higher rates of recurrence [52–54]. While some studies have found this to be true regardless of the approach (open, laparoscopic, or percutaneous) [53], other studies have found worse local tumor recurrence and disease-free survival in percutaneously treated patients compared with open cases [55]. Prognostic factors that contribute to overall outcome include node status of the primary CRC resection, synchronous versus metachronous disease, number and size of lesions, margin status of resected hepatic lesions, CEA levels, the presence of extrahepatic disease, satellite lesions and systemic treatment [51]. RFA is an operator-dependent procedure, whether open, laparoscopic, or percutaneous, and requires careful technique and patient selection to achieve optimal outcomes.

### 3.3. Laparoscopic LR, Hand-Assisted Laparoscopic Surgery (HALS), and Hybrid Approaches

Although initial laparoscopic liver surgery was typically limited to wedge resections of benign lesions that were easily accessible [8, 9, 56], more advanced liver resections are now performed laparoscopically, including totally laparoscopic right, left, and central hepatectomies, extended right and left hepatectomies, and posterosuperior resections [57–63]. 

**Figure 1:**
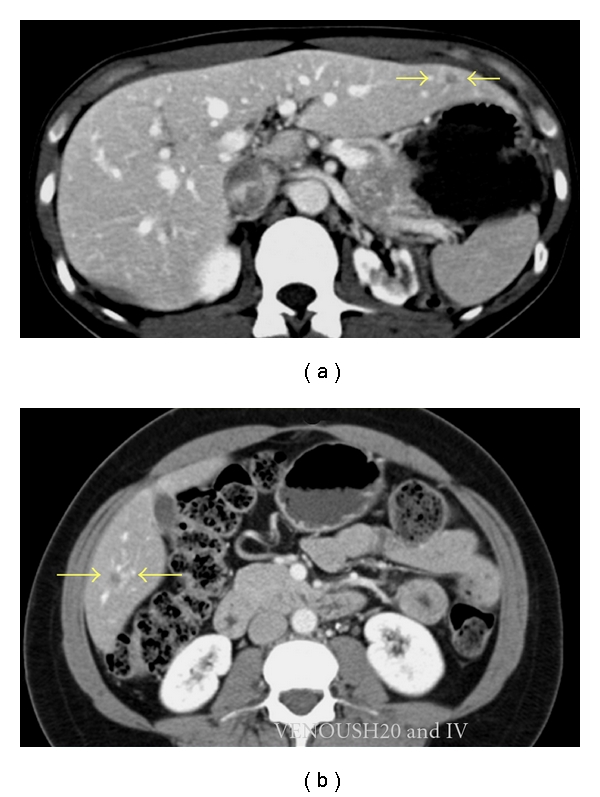
CRLMs accessible for straightforward MILR. (a) A hypodense CRLM in segments 2/3; (b) A hypodense CRLM in segment 6.

Many surgeons wishing to perform MILR begin a stepwise process of beginning with easy minor resections (Figure 1), later using a hand port [64–67] or hybrid technique [68] for complex resections before finally attempting totally laparoscopic major resections. However, even when the dissection and transection of the liver is totally laparoscopic in cases of extended hepatectomies, the specimen is large and requires an incision for removal [58, 59]. This incision is often as long as that required for a hand port, which is one reason that many have advocated for maintaining—and in some cases returning to—a hand-assisted as opposed to a totally laparoscopic approach [66, 67]. The main advantages of HALS are tactile feedback, including ability to palpate the liver, tumor, and nodes, facile liver mobilization and retraction, quick and easy hemostasis with digital compression in cases of unexpected hemorrhage, and the multiple additional uses of the hand-port incision, including placement of additional instruments and removal of the specimen. 

The hybrid technique, combining the relatively basic laparoscopic skills of liver mobilization with the more advanced but open dissection of the liver hilum and transection of the liver parenchyma through a minilaparotomy, the small size of which is made possible by the laparoscopic mobilization [68], is another effort to shorten the learning curve (see below) associated with MILR, thereby increasing the numbers of patients who may benefit from MILR. The hybrid approach, like the HALS approach, is meant to combine the advantages of laparoscopic surgery (decreased postoperative pain and improved cosmesis, largely due to the avoidance of a subcostal incision) with the safety, ease, and accessibility provided in open procedures.

### 3.4. Robotic Liver Resection

Robotic surgery overcomes certain limitations encountered with the laparoscopic technique, such as 2-dimensional images and linear instruments, and offers clear advantages such as 3-dimensional imaging, tremor filtration, higher magnification, and articulating instruments with seven degrees of freedom. These advantages allow improved visualization and improved surgeon dexterity for fine movements, especially intracorporeal suturing. 

Disadvantages inherent in the complex robotic technology include significant expense of the acquisition and maintenance of a robotic system, longer operating times, a significant learning curve to becoming proficient with the setup and utilization of the instruments, a loss of tactile feedback, and the requirement of a skilled assistant, as both the assistant and console surgeon perform integral parts of the hepatic transection [69, 70]. 

The worldwide experience with robotic LR is somewhat limited with approximately 100 reported cases, but the approach appears to be safe in experienced hands [71–74]. The largest series from Giulianotti et al. [71] reported 70 patients, 66 of whom underwent successful robotic resection and 16 of whom had colorectal metastases (4 minor resections and 12 major). The average surgical margin for colorectal metastases was 25 mm and the overall morbidity was 21% with a mortality of 0% and a median length of stay (LOS) of 7 days [71]. Two smaller series of robotic hepatectomies for CRLM and other diagnoses Berber et al. [73] and Ji et al. [74] appear to have similar outcomes with 9 patients and 13 patients, respectively. The margins were not compromised in either group, with morbidity ranging from 7.8 to 11%, with no mortality [73, 74]. Even very complex cases, such as single-stage, combined liver and colon resection for synchronous CRLM, have been performed with robotassisted laparoscopy [75].

### 3.5. Laparoscopic Placement of Hepatic Artery Infusion (HAI) Catheter

Given that a number of studies suggest that regional (as opposed to systemic) chemotherapy may improve the response CRLM and possibly improve survival in patients with resectable and unresectable CRLM [76–80], the principle rational for HAI catheter is that CRLMs derive 80% to 100% of their blood supply from the hepatic artery, as opposed to the portal vein [81]. This allows for a high concentration of chemotherapeutic agents to be delivered directly into the tumors, with maximal effect on the metastatic lesion, minimal parenchymal and systemic toxicity, and minimal loss of activity due to the first-pass hepatic extraction [82]. The laparoscopic approach provides a minimally invasive way to obtain such an access to the hepatic artery by avoiding the morbidity and mortality associated with standard laparotomy procedures. Multiple studies have shown that laparoscopic HAC placement is a feasible and safe procedure [83–85].

### 3.6. Transarterial Embolization, Chemoembolization, and Radioembolization

Hepatic artery infusion is only one of several ways to deliver therapies transarterially to the liver, thereby minimizing systemic toxicities. Other transarterial therapies include transarterial embolization (TAE), transarterial chemoembolization (TACE), and intra-arterial radiotherapy (IART), among others, all of which share with HAI the advantages associated with delivery of therapy via the hepatic arterial system. There is not an overabundance of high-quality data supporting either TAE or TACE over the other, and, when compared, both have produced median survival results of 8–12 months [86, 87]. Nevertheless, since TACE provides two therapies in one—both an ischemic and a toxic insult to the CRLM—it is generally preferred. Albert et al. recently evaluated 121 patients undergoing 245 TACE treatments and found a 27-month overall median survival using cisplatin, doxorubicin, mitomycin C, ethiodized oil, and polyvinyl alcohol particles [88]. Neither TACE nor TAE is as well studied as the treatment of CRLM compared with hepatocellular carcinoma, and more data are needed to define this role.

Similarly, IART is the embolization of a radiation-emitter into the hepatic arteries feeding the CRLM. The only FDA-approved agent is yttrium-90, a radioisotope that emits high-energy beta-particles, which was recently compared in a small phase-I study with FOLFOX for unresectable CRLM [89]. A radiographic partial response was observed in 90% (18/20) of patients and stable disease in 10% (2/20), with a progression-free survival of 9.3 months [89]. As with TAE and TACE, a lack of high-quality data exists, as evidenced by a recent Cochrane review [90] that included only one randomized study: van Hazel et al. [91] compared IART plus systemic chemotherapy and systemic chemotherapy alone and found significantly longer progression-free survival following IART. As with TACE and TAE, more data are needed to improve decision-making in patients with advanced CRLM.

## 4. Comparison of OLR and MILR for CRLM

### 4.1. Oncologic Outcomes

**Figure 2:**
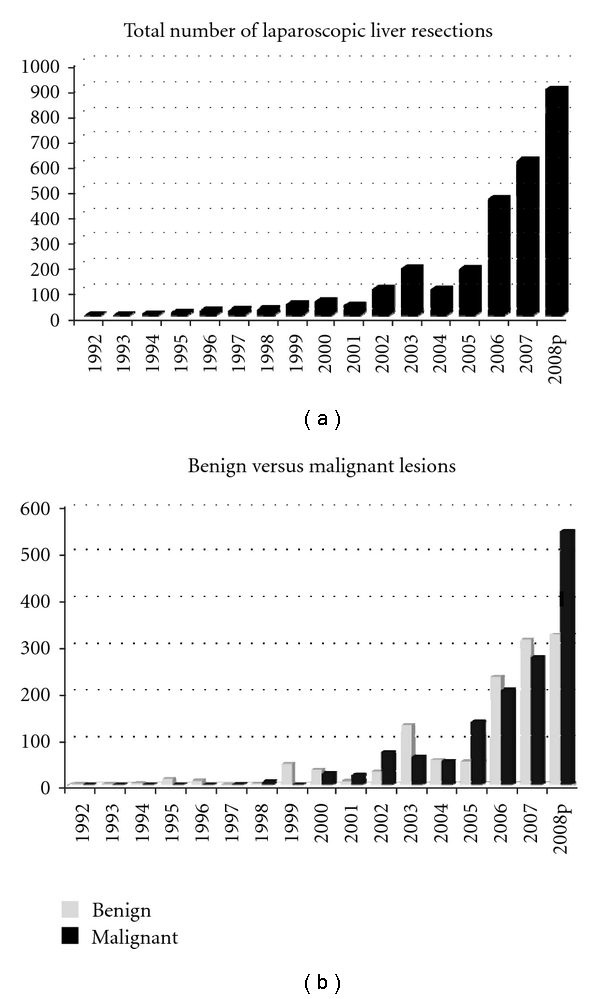
Increase in reported MILRs over time (2002–2008 (partial)). Reproduced from Nguyen et al. Annals of Surgery 2009; 250 : 831 [92].

The treatment of choice for CRLM is surgical resection. As minimally invasive techniques, surgeon familiarity with those techniques, and minimally invasive instruments—particularly coagulation and stapling devices—have improved, the number of patients undergoing MILR has increased exponentially in the recent years (Figure 2 [92]). Although early series reported resections that were typically minor and performed for benign disease [93], increased experience has lead to more aggressive MILR including malignant lesions and major hepatic resections. Early criticism of MILR was aimed at feared inability to maintain oncologic integrity. Indeed, similar concerns were weighed against laparoscopic colon resection until randomized trials supported equivalent safety, negative-margins status, and disease-free survival [94, 95]. A large and growing global experience (but no randomized controlled trials) suggests that laparoscopic resection is safe and effective for the management of liver lesions while maintaining oncologic integrity [64, 65, 92]. 

In the absence of randomized controlled trials comparing MILR with OLR, several observational studies have compared these modalities, including cohort studies, case-controlled studies, and intention-to-treat studies [96–103]; although they show similar rates of complications and survival, many of these contain mixed patient populations including benign and various malignant histologies. Nguyen and colleagues reviewed all laparoscopic resections performed exclusively for CRLM from 2000 to 2008. They identified 109 cases, 97% of which were completed laparoscopically with a 94% negative-margin rate [104]. Forty-five percent of patients underwent a major (≥3 segments) hepatectomy, with no mortalities and a 12% complication rate; actuarial survival at 1, 3, and 5 years was 88%, 69%, and 50%, respectively [104], although no comparison to an open cohort was made. Castaing et al. compared matched cases of laparoscopic and open hepatectomy performed for CRLM and reported comparable 5-year survival rates of 64% and 56% for laparoscopic and open LR, respectively [105], thus supporting comparable oncologic outcomes. While the majority of the laparoscopic resections reported in the literature are minor (<3 segments), Dagher and colleagues studied a series of 210 major hepatectomies from 5 institutions, with 114 for malignant disease [106], reporting a mortality of 1% (non-liver-related) and an overall morbidity of 22% (8.1% liver-related: 6.2% bile leak, 1.4% ascites, and 0.5% hemorrhage). Positive margins were identified in only 3 patients (2.6%) [106], which compares favorably to the rate of margin positivity in OLR for CRLM which has had a wide variance from 5 to 24% [107–109].

### 4.2. Caveats

Although margin status and oncologic outcomes appear favorable these results should be interpreted with caution, since selection bias is inherent in nonrandomized studies and patients deemed laparoscopic candidates may represent a different and more favorable group. In the Castaing series, for instance, the operative details are variable, with vascular clamping occurring in 92% of the open versus 17% in the laparoscopic group [105]. An interesting study performed by Welsh et al. reviewed all patients who underwent OLR for CRLM and divided them into 2 groups, one suitable for MILR and another deemed best candidates for OLR. Even though all resections were done open, those identified as MILR candidates had a lower positive-margin rate (4.5% versus 15%) and better 5-year survival (44% versus 37%) [110].

Randomized data comparing MILR and OLR are, unfortunately, unlikely to be obtained because of difficulty in defining end points (safety versus cost versus efficacy), none of which were deemed good end points for such a trial in the recent international consensus Louisville Statement [64], because of heterogeneity of the patient population and because of the length of time needed to accumulate enough patients. The Louisville Statement did support a prospective registry with preoperative enrollment, the creation of which would help track the dissemination of procedures aimed at improving patient safety [64]. Although initial nonrandomized data suggest that MILR is likely a safe, oncologically sound means of managing CRLM, ongoing critical review, multidisciplinary teams and participation in prospective data collection or trials will continue to define optimal approach to CRLM.

### 4.3. Cost and LOS

Examination of cost has been reported by several groups in retrospective series. In a subgroup analysis from the University of Louisville, Buell et al. retrospectively reviewed and compared 29 laparoscopic and 34 open resections, finding significant actual cost savings for a laparoscopic approach to major resections ($21,131 versus $36,821; *P* < 0.01) before, but not after, adjustment for changes in Diagnosis-Related Group coding ($25,457 versus $23,691; *P* < 0.2) [111]. In a retrospective comparison of open versus laparoscopic left lateral segmentectomy, Vanounou et al. at the University of Pittsburgh used deviation-based cost modeling to analyze open and laparoscopic liver resections (for both benign and malignant diseases) and found that the overall LOS was 2 days shorter for the laparoscopic cases, which was associated with a per-patient cost reduction of $2939 compared to open cases [112]; when only malignant cases were considered, however, that cost reduction was attenuated at $1527 per patient. 

Although both the Louisville and the Pittsburgh studies showed significantly shorter LOS for MILR versus OLR, other data, derived from comparing standard recovery pathway versus a fast-track recovery pathway following OLR, have found a similar reduction in LOS (2 days) following fast-track recovery [113]. Indeed, it has been argued [110, 114, 115] that LOS is determined more by extent of resection than by operative approach and may be as little as 2–4 days with either laparoscopic or open approach.

## 5. Future Directions and Controversies

### 5.1. Training and Credentialing

Broad surgical experience, corroborated by many studies [116–118], demonstrates the learning curve for MILR. Simillis et al., for instance, showed that only in studies published after 2003 and in studies including ≥20 laparoscopic procedures did the operative blood loss, LOS, and complications decrease for laparoscopic liver surgery when compared to open LR [117]. As the number of MILR cases performed increases worldwide at a tremendous rate (Figure 2 [92]) and as the MILR community increasingly recognizes the infeasibility of performing randomized trials of MILR [64], there have been many calls for an international registry [64, 92] of MILR to maintain a record for monitoring outcomes of efficacy and patient safety, as has already been achieved for natural orifice transluminal endoscopic surgery [119], an experimental modality much more widely used for cholecystectomy (85% of all NOTES procedures) [119] than for MILR [120, 121].

Training and credentialing of MILR are currently performed at the local level and left to individual institutions. At a minimum a strong combined expertise in major laparoscopic surgery and advanced hepatobiliary techniques, including knowledge and skill in the use of intraoperative ultrasound, is required of a surgeon who wishes to begin the learning curve for MILR [64, 117]. Currently, however, there are no clear, widely accepted criteria that define this required expertise [64]. A certification process for MILR has also yet to be defined and the American Hepato-Pancreato-Biliary Association does not currently require a specific number of MILR cases for the training of hepatobiliary surgeons [122]. Many surgeons, however, recognize the need for a systematic progression from basic to advanced laparoscopic and hepatobiliary skills then to combined advanced laparoscopic and hepatobiliary skills, and some have published their experience with starting an MILR program from scratch [123].

### 5.2. Learning Curve Effect

1. The learning-curve effect has not been studied extensively in laparoscopic liver surgery. Numerous single-institution series have shown an improvement in outcomes when the latter experience is compared to the early experience [124, 125]. The first detailed analysis evaluating the learning curve effect in MILR was published by Vigano et al. in 2009, revealing significantly improved operative time, conversion rate, blood loss, morbidity, and hospital stay progressively over time as experience and volume increased, despite an increase in operative complexity over time [126]. The shape of the learning curve was, not surprisingly, similar to those reported regarding laparoscopic colectomy [127]. These results suggest that MILR is reproducible in selected high-volume centers, by surgeons with advanced laparoscopic and hepatobiliary training.

### 5.3. Combined Liver and Lung Metastases

1. Although the liver is the most common site of colorectal metastasis [3], another 3.5% of patients with colon—and 11.5% of patients with rectal—cancer will develop lung metastasis [128], most of whom have both liver and lung diseases. Although early (prior to 2001) studies of curative-intent metastasectomy in patients with combined hepatic and pulmonary colorectal metastases found widely variable 5-year survival rates, ranging from 11% to 44% [129–132], more recent retrospective studies have produced improved 5-year survival rates as high as 64% [133–137] in patients with colorectal pulmonary metastases. Although fewer data are available regarding minimally invasive treatment of pulmonary—as compared to hepatic—colorectal metastases, large recent series have shown that pulmonary metastasectomies may be performed with minimally invasive video-assisted thoracic surgery [137]. Another, even less invasive option is RFA of pulmonary metastases. Yamakado et al. recently studied a series of 78 patients with 198 colorectal lung metastases treated with RFA, reporting a 5-year and median survival of 35% and 38 months, respectively [138].

### 5.4. Combined Liver and Peritoneal Metastases

1. Large series of patients with CRC have revealed that 4% to 19% of patients have colorectal carcinomatosis at the time of the CRC resection or in follow up, although as many as 80% of patients who die of CRC have developed carcinomatosis by the time of their death [139, 140]. In a review of nearly 3000 cases of CRC in Singapore, 349 (13%) had carcinomatosis, 61% (214) of whom had disease that was synchronous—and 39% (135) metachronous—with their initial presentation of CRC [139]. The proportions of CRLM patients with no carcinomatosis, with metachronous carcinomatosis, and with synchronous carcinomatosis were 10%, 33%, and 42%, respectively, respectively [139]. An increasing body of data [141], including randomized controlled data [142], suggests that complete surgical eradication of metastatic peritoneal disease with cytoreduction and hyperthermic intraperitoneal chemotherapy (HIPEC) is beneficial to selected patients with colon cancer with carcinomatosis, with 5-year survival rates >50% recently reported [143].

Although the presence of CRLM is often considered a contraindication to cytoreduction and HIPEC, and similarly the presence of carcinomatosis a contraindication to LR, several recent studies [144–147] have evaluated the combination of cytoreduction, HIPEC, and liver resection as an aggressive emerging option for highly selected CRC patients with both CRLM and peritoneal carcinomatosis. Elias et al. [147] evaluated a series of 24 such patients with a mean Peritoneal Cancer Index (PCI) [148] of 8.6 (range: 2–25), half of whom underwent a major hepatectomy, and reported one postoperative death, a morbidity of 58%, and an overall 2-year survival of 61%. Patients with ≥3 CRLM had significantly worse survival compared with patientS who had <3 CRLM [147]. More recently Chua et al. [144] evaluated a series of 16 patients with both CRLM and carcinomatosis treated by combined cytoreduction, HIPEC, and liver resection and reported a 2-year survival of 65%; neither survival nor perioperative factors such as morbidity and LOS were different compared with patients who had isolated carcinomatosis without CRLM, although those with both CRLM and carcinomatosis had significantly lower PCI compared with patients with isolated carcinomatosis [144]. Most patients with CRLM and peritoneal carcinomatosis are not, however, currently candidates for aggressive surgical resection of their CRLM and these emerging data should be interpreted with caution.

Minimally invasive techniques have been used to perform cytoreduction to palliate metastatic disease to the ovaries [149], to completely remove primary ovarian carcinomas with limited peritoneal dissemination [150], and to do staged laparoscopic HIPEC following open cytoreduction [151]. The results of an ongoing protocol on laparoscopic cytoreduction and HIPEC in patients with limited peritoneal dissemination appear promising and compare favorably to those patients having an open cytoreductive procedure [152], suggesting that combining MILR and minimally invasive cytoreduction and HIPEC is on the horizon.

## 6. Summary

The number of cases of MILR performed in the world has increased exponentially in recent years, and many centers are now performing major, complex resections. In the absence of randomized trials comparing OLR and MILR, which is not likely obtainable, nonrandomized data suggest that MILR produces similar or improved morbidity, mortality, LOS, and cost compared with OLR, although significant selection bias exists. Because randomized data will be difficult or impossible to obtain, patient registries should be used to track safety and efficacy outcomes. Training of surgeons similarly should become more formalized, including an independent process for surgeon credentialing.
